# The wound healing response after implantation of a drug-eluting stent is impaired persistently in the long term

**DOI:** 10.1007/s00380-015-0676-y

**Published:** 2015-05-05

**Authors:** Takahisa Nasuno, Michiaki Tokura, Michiya Kageyama, Shigeru Toyoda, Masashi Sakuma, Takaaki Komatsu, Isao Taguchi, Shichiro Abe, Teruo Inoue

**Affiliations:** Department of Cardiovascular Medicine, Dokkyo Medical University, 880 Kitakobayashi, Mibu, Tochigi 321-0293 Japan; Department of Cardiovascular Medicine, Koshigaya Hospital, Dokkyo Medical University, 2-1-50 Minamikoshigaya, Koshigaya, Saitama 343-8555 Japan

**Keywords:** Drug-eluting stent, Wound healing, Inflammation, Neoatherosclerosis

## Abstract

A 70-year-old man underwent stent implantation for right coronary artery (RCA) lesions with a bare metal stent (BMS) and two sirolimus-eluting stents (SES). However, as both the BMS and SES stented sites developed restenosis after 13 months, he underwent target lesion revascularization using directional coronary atherectomy (DCA). On histopathology, the restenosis lesion at the SES-deployed site showed greater inflammation and less re-endothelialization than that at the BMS-deployed site. Three months later, the SES-deployed site developed a second restenosis, in which paclitaxel-eluting stents (PES) were implanted (PES-in-SES), while the BMS-deployed site was restenosis free. Five years later, restenosis was absent in these RCA lesions. However, by optical coherence tomography and/or coronary angioscopy, the PES-in-SES site in the RCA showed poor neointimal coverage over the stent struts and yellowish neointima, suggesting lipid-rich neoatheroma formation, whereas at the BMS site appropriate white neointima formation was observed. Drug-eluting stents still have problems of persistent inflammation, inappropriate neointima formation, and neoatherosclerosis. Although we are now in the era of second generation DESs in which better stent performance would be promising, we should remember that we are obliged to continue to follow-up all patients in whom first generation DESs such as SES or PES have been placed.

## Introduction

We have previously described a patient who developed diffuse restenosis in the right coronary artery, where a bare metal stent (BMS) and two drug-eluting stents (DES) had been deployed sequentially 13 months before. We compared the differences in pathological features between the restenosis lesion at the DES site and those at the BMS site, using specimens obtained by directional coronary atherectomy (DCA). We observed delayed re-endothelialization and prolonged inflammation in the DES lesion relative to the BMS lesion [1]. Thereafter, we carefully followed this case for 5 years.

In the present report, we describe our latest findings at the stent sites in this case, in which we observed neointima formation over the stent strut by optical coherence tomography (OCT) and coronary angioscopy (CAS).

## Case report

**Fig. 1:**
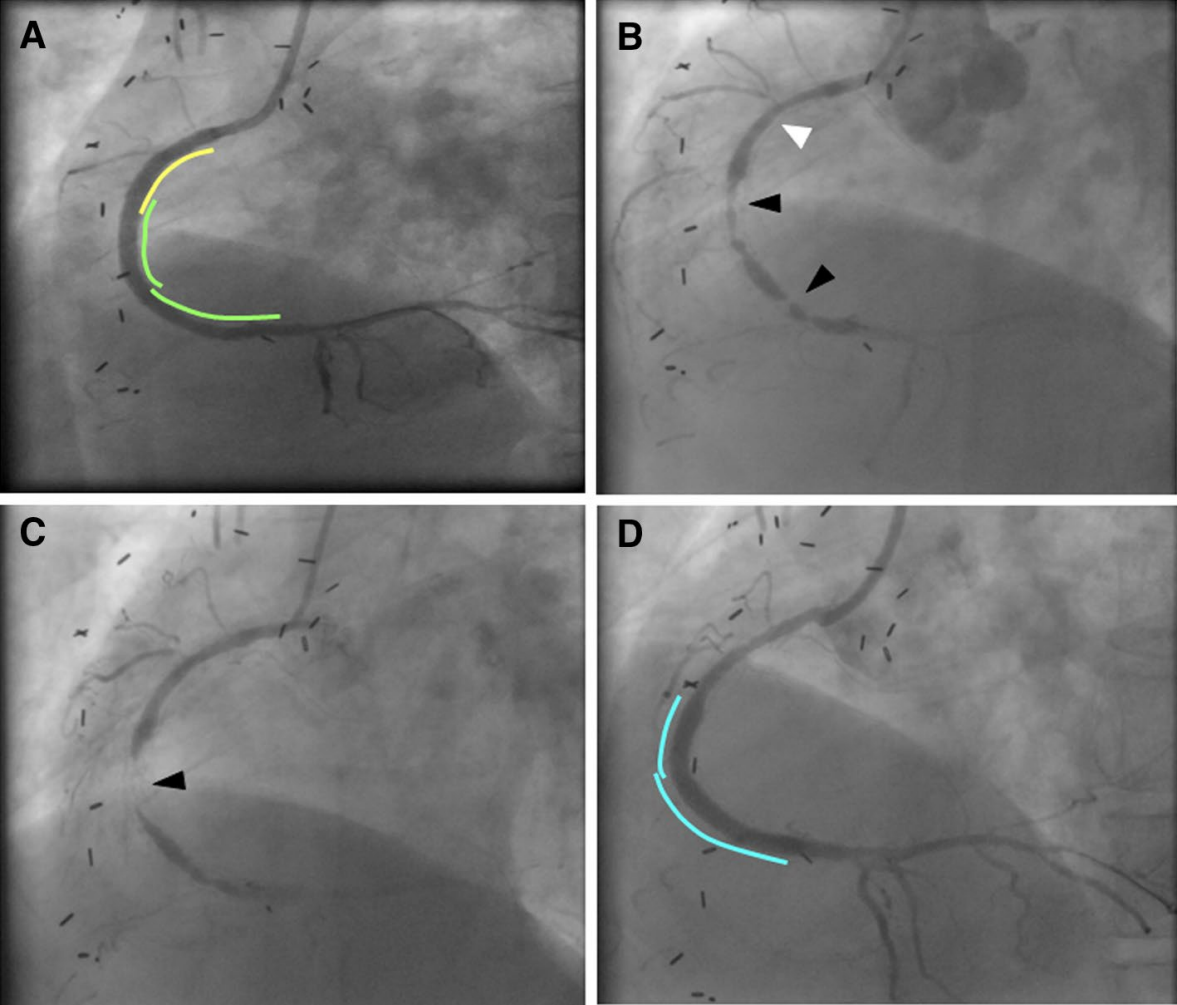
**a** A bare metal stent (BMS) (*yellow line*) and two sirolimus-eluting stents (SES) (*green lines*) were deployed at the proximal and distal portions of the right coronary artery (RCA), respectively, with sequential edge to edge overlap to achieve full coverage of the longitudinal plaque. **b** Thirteen months later, in-stent restenosis had developed at both the BMS-deployed site (*white arrow*) and the SES-deployed site (*black arrows*). **c** Three months after the target lesion revascularization by balloon angioplasty following directional coronary atherectomy, a second restenosis had developed at the SES site alone (*black arrow*). **d** A pair of paclitaxel-eluting stents (PES) were implanted in the second restenosis site (PES-in-SES) (*blue lines*)

**Fig. 2:**
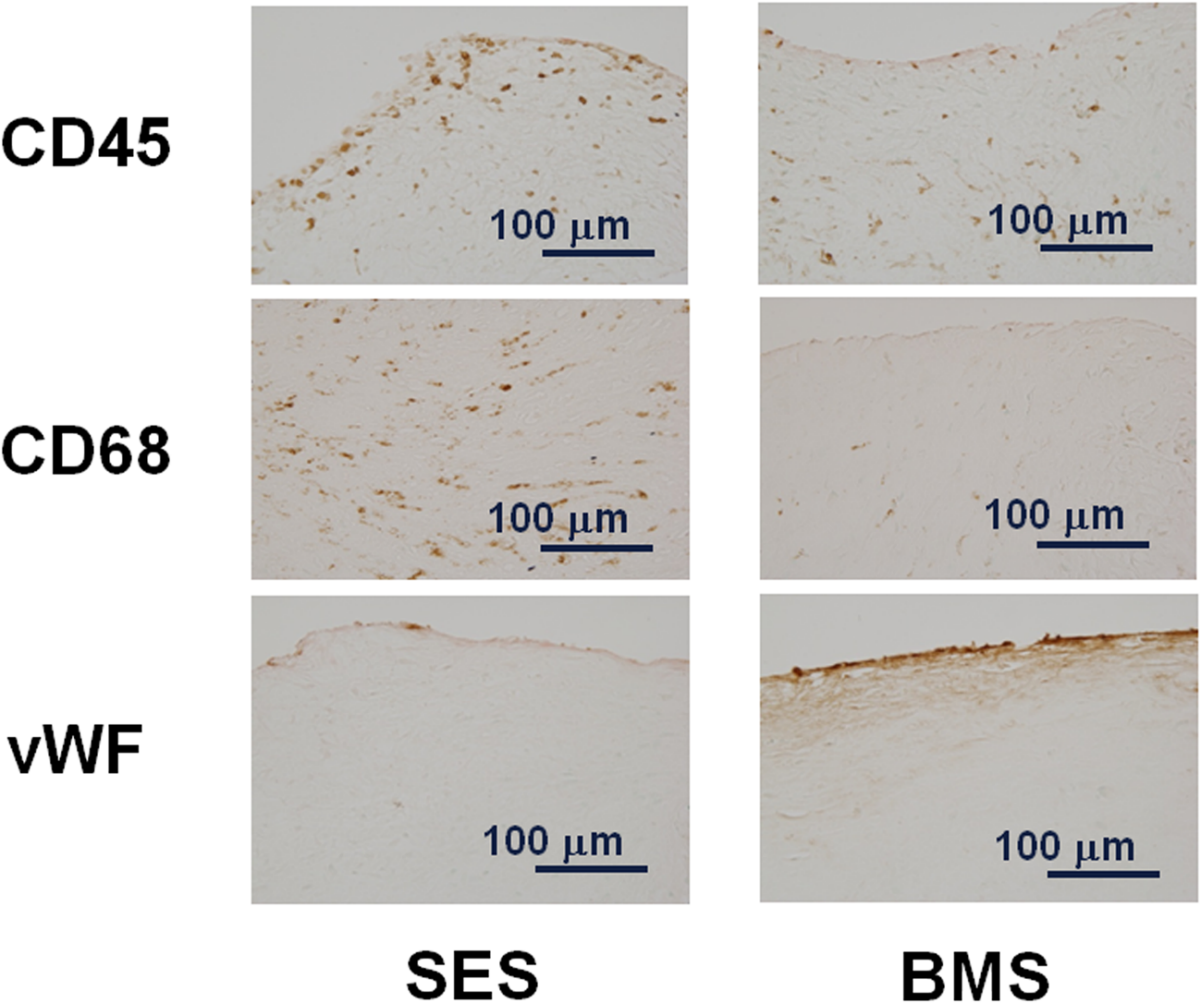
Immunohistochemistry of tissue samples at restenosis lesions of the BMS-deployed site and the SES-deployed site, obtained by the directional coronary atherectomy (DCA) 13 months after stent deployment. Dense accumulations of CD45+ lymphocytes and CD68+ macrophages were present in the restenosis lesions at the SES site, but the accumulation of inflammatory cells was slight in those at the BMS site. Endothelial cells as stained with von Willebrand factor (vWF) were present in the restenosis lesions at the BMS site, but absent from those at the SES site

**Fig. 3:**
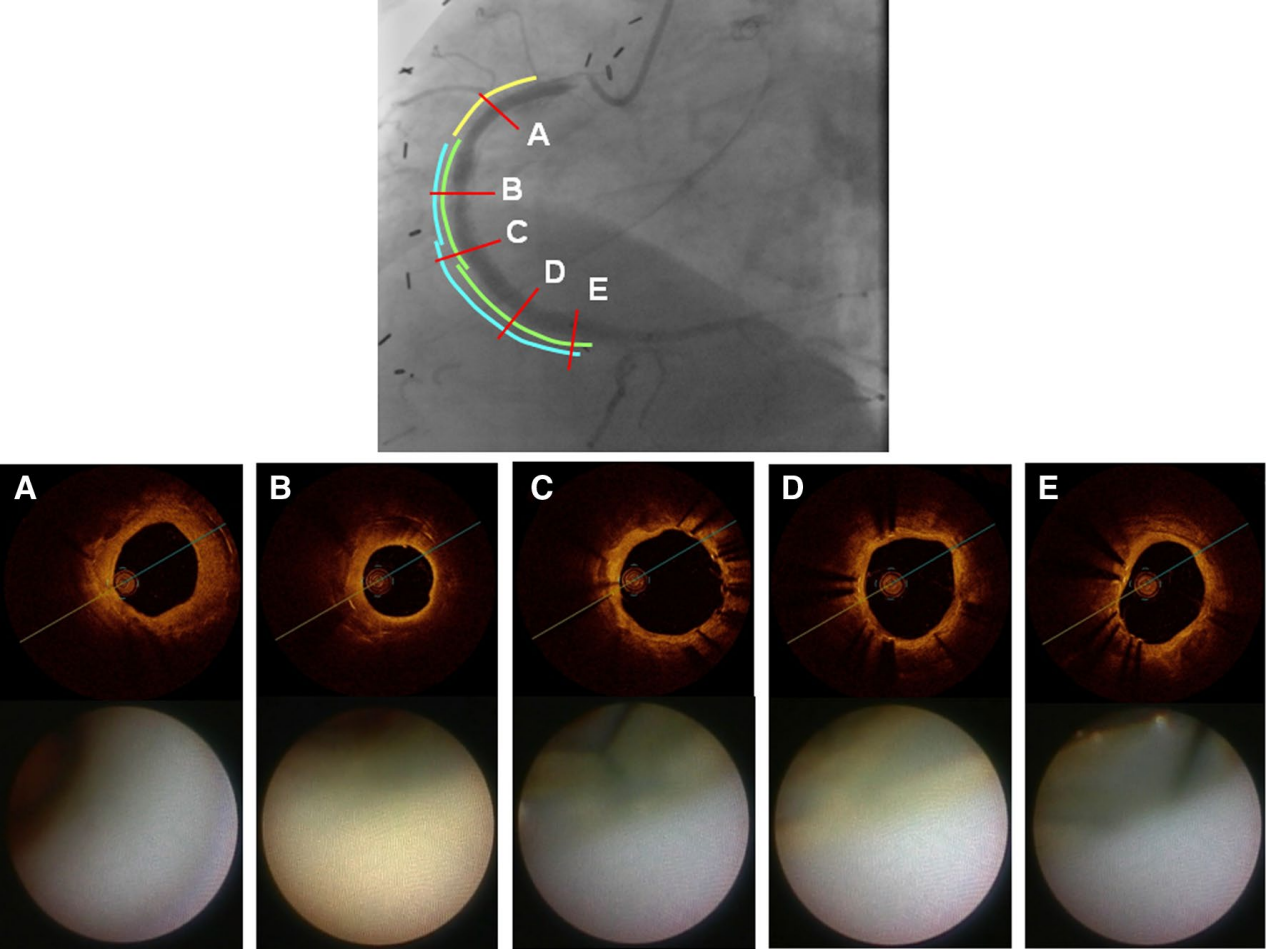
The latest findings in the RCA lesions by coronary angiography (*upper panel*), optical coherence tomography (OCT) and coronary angioscopy (CAS) (*lower panel*). In the BMS-deployed site, stent struts were well covered with homogeneous uniformly thick, white colored neointima (**a**). In the proximal PES-in-SES site, a mild late catch-up phenomenon was observed angiographically, attenuated neointima was observed by OCT, and the neointima was strongly yellowish on CAS (**b**). In the middle portion of the PES-in-SES site, the stent struts were well covered by neointima, which was mildly yellowish on CAS (**c**, **d**). In the distal portion of the PES-in-SES site, neointima was thin on OCT, and several stent struts were clearly visible by CAS (**e**)

A 70-year-old man with poorly controlled diabetes mellitus, who had a history of angina pectoris and coronary artery bypass surgery for three-vessel coronary artery disease, underwent percutaneous coronary intervention (PCI) for a native chronic total occlusion of the right coronary artery (RCA), as the graft for the RCA had become obstructed. A BMS and two sirolimus-eluting stents (SES) were deployed at the proximal to distal portion, respectively, with sequential edge to edge overlap to achieve full coverage of the longitudinal plaque (Fig. 1a). Thirteen months later, in-stent restenosis had developed at both the BMS-deployed site and the SES-deployed site (Fig. 1b). DCA was performed for target lesion revascularization (TLR) under intravascular ultrasound guidance for both regions of post-BMS and post-SES. The site, where the BMS and SES were overlapping, was not treated by the DCA, because it was confirmed by the IVUS observation that the restenosis lesion was absent at the overlapping site. Immunohistochemistry of tissue samples obtained from the DCA showed dense accumulations of inflammatory cells such as T lymphocytes and macrophages in the restenosis lesions at the SES site, but not in those at the BMS site. It also revealed that, while endothelial cells were absent from the restenosis lesions at the SES site, they were present in those at the BMS site (Fig. 2) [1]. The TLR was successfully performed by balloon angioplasty following the DCA. However, 3 months later the patient complained of angina, and coronary angiography showed a second restenosis at the SES site alone (Fig. 1c). Therefore, a pair of paclitaxel-eluting stents (PES) were implanted in the second restenosis site (PES-in-SES) (Fig. 1d). Five years later, he was readmitted with unstable angina caused by a new lesion in the diagonal branch. Restenosis was not evident in the stented sites of the RCA lesions. When performing PCI for the diagonal branch lesion, we examined the neointima at the stent sites in the RCA by OCT and CAS. In the BMS-deployed site, the stent struts were well covered with homogeneous uniformly thick, white colored neointima (Fig. 3a). In the proximal PES-in-SES site, which was the most severe second restenosis site post-SES stenting, a mild late catch-up phenomenon was observed angiographically, attenuated neointima was observed by the OCT, and the neointima was strongly yellowish on CAS (Fig. 3b). In the middle portion of the PES-in-SES site, stent struts were well covered by neointima, which was mildly yellowish on CAS (Fig. 3c, d). In the distal portion of the PES-in-SES site, the neointima was thin by OCT, and several stent struts were clearly visible by CAS (Fig. 3e).

**Fig. 4:**
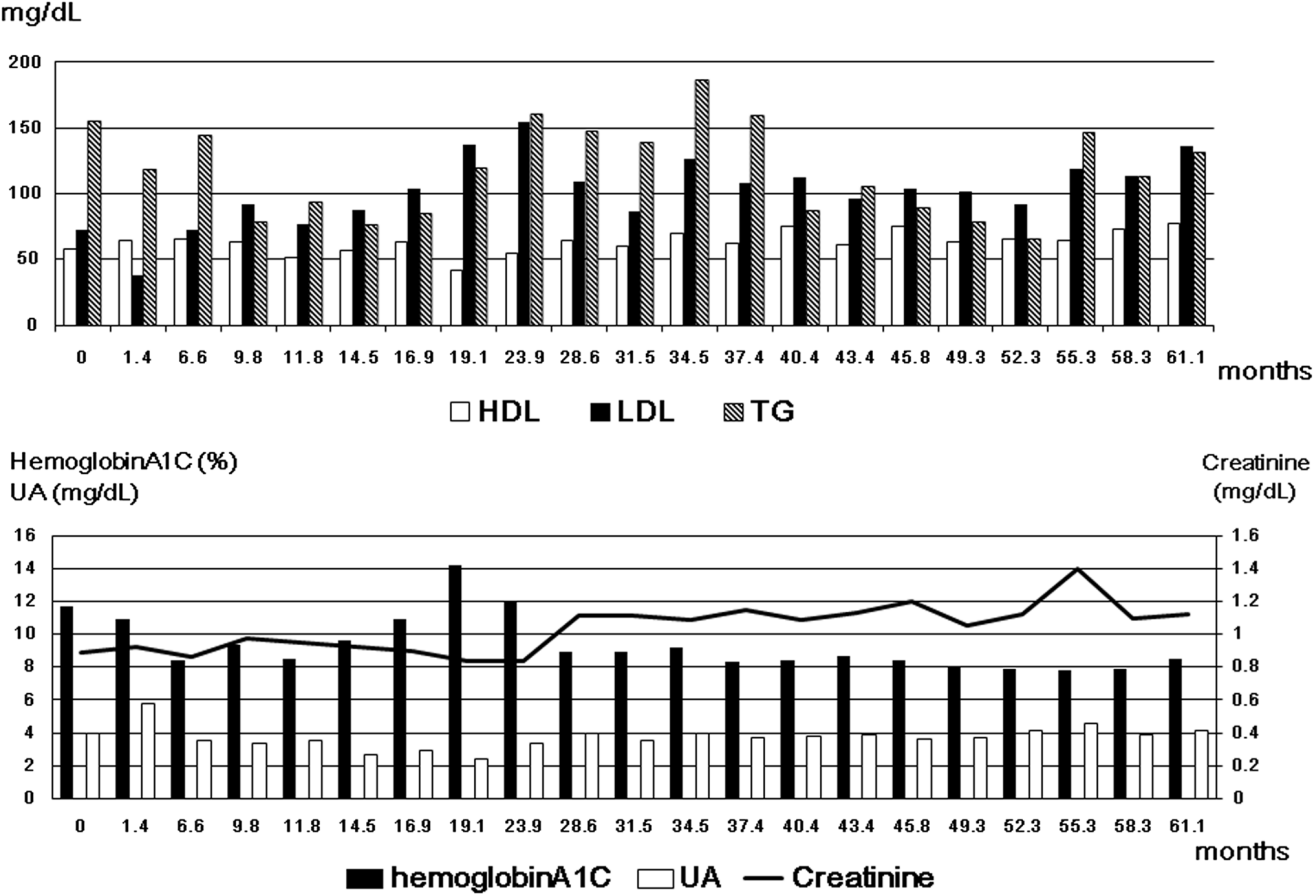
Course of risk factor parameters during 5 years from the final percutaneous coronary intervention. Dyslipidemia was well controlled (*upper panel*). Although serum uric acid was maintained at low concentrations, diabetes was not treated optimally, and serum creatinine progressively increased (*lower panel*)

During 5 years from the final PCI to the PES-in-SES procedure, dyslipidemia was not strictly controlled (Fig. 4, upper panel) (HDL-cholesterol: 63.2 ± 8.2 mg/dL; LDL-cholesterol: 101.9 ± 26.1 mg/dL; triglycerides: 118.1 ± 34.5 mg/dL). Although serum uric acid was maintained at low levels (3.7 ± 0.7 mg/dL), diabetes was poorly controlled (hemoglobin A1c: 9.3 ± 1.6 %), and the serum creatinine concentration gradually increased (Fig. 4, lower panel).

## Discussion

The patient underwent repeated revascularization. Since the graft for the RCA became occluded, a BMS and two SESs were sequentially implanted in the native RCA. However, 13 months later restenosis had developed in both the BMS-deployed and SES-deployed sites. We selected DCA for TLR at both sites, because this patient had poorly controlled diabetes, and thus, less injury by applying DCA technology would have been promising. Surprisingly, the histopathological features of the restenosis lesions, samples of which were taken using DCA, were quite different. The SES-restenosis lesions showed less endothelial regeneration and a greater inflammatory reaction than the BMS-restenosis lesions, as mentioned in our previous case report [1]. We have also observed similar histopathological findings in several other cases [2].

After the first revascularization, restenosis did not occur at the BMS-restenosis site during long-term follow-up of more than 5 years. In the SES-restenosis site, on the other hand, restenosis recurred after 3 months. Finally, this site was treated with the PES-in-SES technique, after which these RCA lesions remained free of restenosis for 5 years, although a new lesion in the diagonal branch developed. In the present report, the most important finding is that by OCT and/or CAS the restenosis-free PES-in-SES site in the RCA showed poor neointimal coverage over the stent struts and yellowish neointima, suggesting in-stent lipid-rich neoatheroma formation. Previously neointimal tissue characterization was compared between DESs and BMS using iMap intravascular ultrasound (IVUS) observation. As a result, DES implantation was associated with more-necrotic and less-fibrotic neointimal formation, suggesting the development of in-stent neoatherosclerosis [3]. This finding would be in substantial agreement with our present data. Although the IVUS-based tissue characterization might be useful to assess the in-stent neoatheroma, we believe that OCT and/or CAS observation in our study would have greater advantage to assess neointimal coverage.

Drugs coated on the surface of drug-eluting stents (DES) are potent anti-mitotic and anti-inflammatory agents that strongly inhibit smooth muscle cell proliferation and matrix growth, thereby reducing neointima formation and restenosis [4–6]. Although DES has substantially reduced the problem of restenosis, a separate issue has arisen distinct from restenosis, namely the potential for impairment of re-endothelialization, which can lead to insufficient neointimal coverage and subsequent stent thrombosis, the latter sometimes occurring very late after implantation [7–9]. Impaired re-endothelialization may also delay wound healing and cause a relapse of inflammation when the anti-inflammatory effects of the drugs on the DES have disappeared [10], leading to neoatherosclerosis with vulnerable plaque (i.e., neoatheroma formation) at the stented sites [11–13]. Therefore, the overall performance of a stent should be characterized by the geometric luminal gain acquisition, appropriate neointima formation to achieve sufficient stent strut coverage, and prevention of neoatherosclerosis.

The most important message from this case report is that both the restenosis lesion 13 months after the first PCI and the restenosis-free site 5 years after the final PCI showed strong inflammatory features and less re-endothelialization after DES placement, whereas the BMS placement site did not do so. Since diabetes and dyslipidemia were not treated optimally and renal function progressively deteriorated in this case, it cannot be denied that these factors contributed to the lesion outcomes.

This case demonstrates that although DES can achieve satisfactory geometric luminal gain, the problems of persistent inflammation, inappropriate neointima formation and neoatherosclerosis remain to be overcome. Finally, the SES and PES used in this case are first generation DESs, neither of which is now available. Although we are now in the era of second generation DESs in which better stent performance would be promising [14], we should remember that we are obliged to continue to follow-up all patients in whom first generation DESs have been placed.
